# Real-world characterization of blood glucose control and insulin use in the intensive care unit

**DOI:** 10.1038/s41598-020-67864-z

**Published:** 2020-07-01

**Authors:** Lawrence Baker, Jason H. Maley, Aldo Arévalo, Francis DeMichele, Roselyn Mateo-Collado, Stan Finkelstein, Leo Anthony Celi

**Affiliations:** 1 RAND Corporation, Santa Monica, CA USA; 2 Division of Pulmonary, Critical Care, and Sleep Medicine, Beth Israel Deaconess Medical Center, 330 Brookline Ave., Boston, MA 02215 USA; 3 Center for Healthcare Delivery Science, Beth Israel Deaconess Medical Center, Boston, MA USA; 4 Harvard Medical School, Boston, MA USA; 5 MIT Laboratory for Computational Physiology Harvard–MIT Division of Health Sciences and Technology, Cambridge, MA USA; 6 IDMEC, Instituto Superior Técnico, University of Lisbon, Lisbon, Portugal; 7 Landmark Health, Huntington Beach, CA USA; 8 Division of Endocrinology and Metabolism, Rush University Medical Center, Chicago, IL USA

**Keywords:** Endocrine system and metabolic diseases, Epidemiology, Health services

## Abstract

The heterogeneity of critical illness complicates both clinical trial design and real-world management. This complexity has resulted in conflicting evidence and opinion regarding the optimal management in many intensive care scenarios. Understanding this heterogeneity is essential to tailoring management to individual patients. Hyperglycaemia is one such complication in the intensive care unit (ICU), accompanied by decades of conflicting evidence around management strategies. We hypothesized that analysis of highly-detailed electronic medical record (EMR) data would demonstrate that patients vary widely in their glycaemic response to critical illness and response to insulin therapy. Due to this variability, we believed that hyper- and hypoglycaemia would remain common in ICU care despite standardised approaches to management. We utilized the Medical Information Mart for Intensive Care III v1.4 (MIMIC) database. We identified 19,694 admissions between 2008 and 2012 with available glucose results and insulin administration data. We demonstrate that hyper- and hypoglycaemia are common at the time of admission and remain so 1 week into an ICU admission. Insulin treatment strategies vary significantly, irrespective of blood glucose level or diabetic status. We reveal a tremendous opportunity for EMR data to guide tailored management. Through this work, we have made available a highly-detailed data source for future investigation.

## Introduction

Precision medicine that proposes the customization of medical decision making may be transformative in the intensive care unit (ICU) setting, where the complexity and ambiguity of common illness syndromes and therapeutic responses vary widely from patient to patient. Historically, heterogenous patients have often received one-model-fits-all guideline-based treatment strategies. However, patient sub-phenotypes have now been identified by clinical and biomolecular profiles within syndromes such as sepsis and acute respiratory distress syndrome (ARDS)^1,2^. When these phenotypes are examined in prior clinical trials data, they exhibit strikingly different prognoses and responses to treatment^1,3,4^. Unpacking this heterogeneity may inform future trials and the provision of individualised treatment approaches.

Hyperglycemia remains a commonly encountered complication in the ICU, with decades of conflicting evidence around blood glucose targets and management strategies. Both hyperglycaemia and hypoglycaemia have been linked to increased morbidity and mortality. An early study of patients with myocardial infarction reported increased risk of cardiogenic shock and death among both diabetic and non-diabetic patients when presenting serum glucose was greater than 180 mg/dL^5^. Following this, retrospective studies of broad populations of critically ill patients observed similar increased risk of death with hyperglycaemia, at glucose levels greater than 140–180 mg/dL^5–7^. Thus, interest grew in prospective study of increasingly tighter control of blood glucose levels. In support of this approach, a seminal randomized trial of mechanically ventilated patients, the majority of whom were admitted after cardiac surgery, demonstrated that intensive glucose control (80–110 mg/dL) resulted in decreased mortality^8^. However, a subsequent single-centre study of intensive glucose control for patients admitted to a medical ICU found no difference in mortality^9^. Finally, a large, international randomized study of glucose control in the range of 81–108 mg/dL versus 144–180 mg/dL demonstrated increased mortality from intensive glycaemic control, likely as a result of inadvertent hypoglycaemia^10^. As a result of these studies, current ICU insulin protocols tend to prioritize prevention of harmful hypoglycaemia (glucose < 80 mg/dL), while maintaining glucose levels within the range supported by the most contemporary trial, approximately 140–180 mg/dL.

Our clinical experience suggests that the challenges of glucose management may differ significantly in real-world ICU practice from that of strictly monitored trials. We sought to examine highly-detailed and large scale ICU data to better understand the landscape of glucose management in clinical practice. We hypothesized that: (1) patients have widely varied glycaemic responses to critical illness, (2) this variability results in unpredictable responses to corrective insulin, and (3) as a result of these challenges, hypo- and hyperglycaemia remain common in the ICU despite standardised approaches to management. Finally, we developed and made publicly available a large curated dataset through this investigation. This dataset could be a valuable resource to develop an individualised approach to blood glucose management, which prospective trials may not feasibly address.

## Results

**Figure 1:**
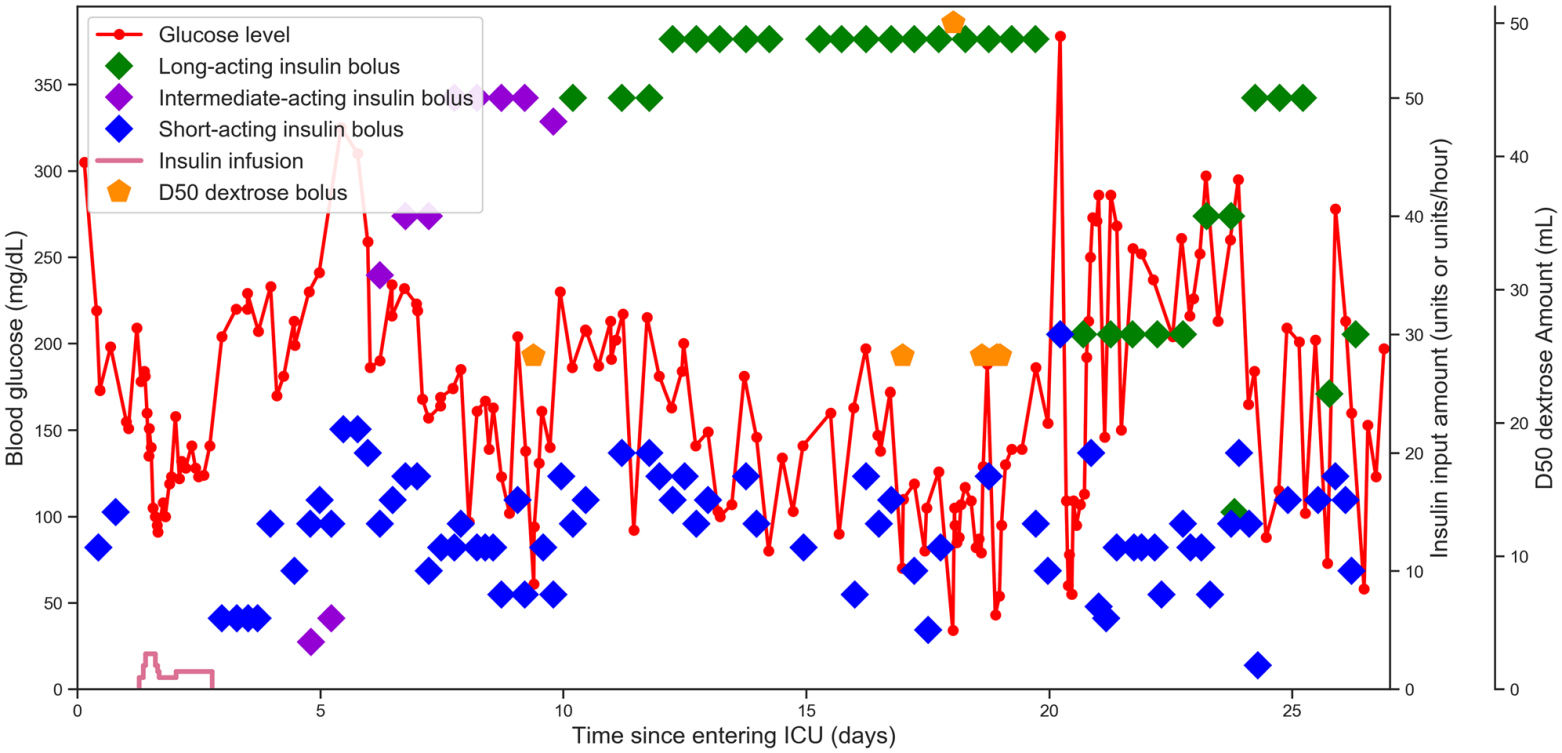
Representative ICU admission. Graph is constructed from electronic data, demonstrating glucose trend over time along with insulin and dextrose inputs.

We included 19,694 admissions, between 2008 and 2012, in our analysis. The median patient age was 66 years, 56% were male, 73% were white, and 24% were diabetic (Table 1). 80.8% of short-acting insulin boluses were successfully matched with a corresponding glucose measurement, providing a detailed longitudinal account of events occurring during each ICU admission (Fig. 1).

**Table 1:** Characteristics of patient cohort.

Patient characteristics at baseline	
Age (median)	65.9
Female (%)	44
Male (%)	56
**Race (%)**	
White	73
Black	10
Hispanic	4
Other	13
**Diabetic status (%)**	
Diabetic	73
Non-diabetic	10
**Admission type**	
Elective	14
Emergency	86
Medical ICU (%)	42
Cardiac ICU (%)	12
Surgical ICU (%)	46
Died in hospital (%)	11

### Frequency of hyperglycaemia and hypoglycaemia

**Figure 2:**
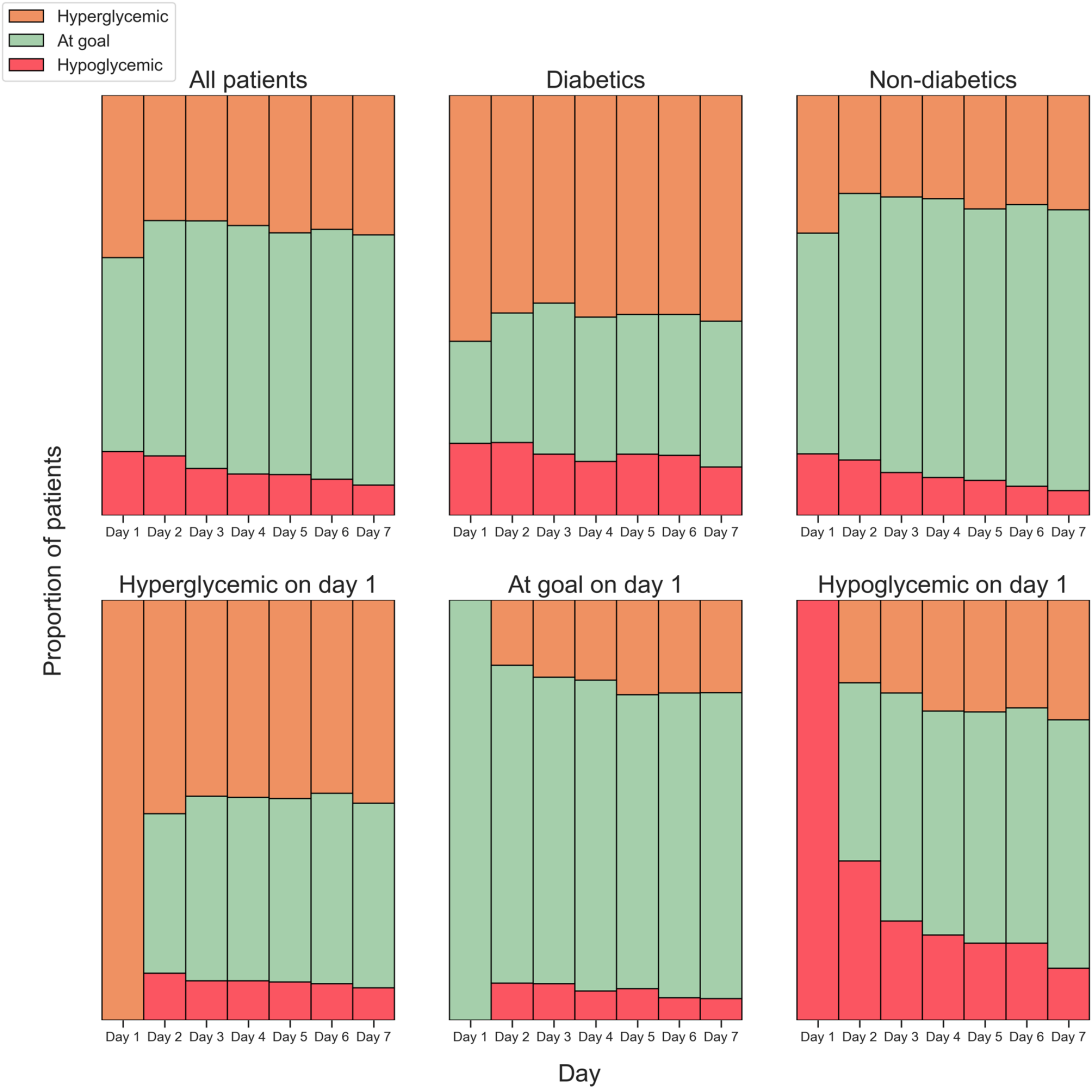
Hyper and hypoglycaemic events by diabetic and first day status. Daily hyperglycaemia (orange, one or more measurements > 180 mg/dL), hypoglycaemia (red, one or more measurements < 80 mg/dL), and euglycaemia (green) among first ICU stays with glucose readings over the first 7 days (n = 2,759). Each plot shows a different group of patients: all patients, diabetics, non-diabetics, those who had a hyperglycaemic event on day 1, those who met glucose targets on day 1, and those who had a hypoglycaemic event on day 1.

Patients enter the ICU with high rates of hyperglycaemia and hypoglycaemia. This commonly persists over the course of their stay, especially for those who were initially hyperglycaemic. Those who were initially hypoglycaemic were at increased risk for further hypoglycaemia, but this risk diminished over the course of their stay (Fig. 2). Diabetics were more likely to have hyperglycaemic events than non-diabetics.

On the day of ICU admission: 28.6% of diabetic and 9.3% of non-diabetic patients had average daily blood glucose in the hyperglycaemic range; 2.0% of diabetic patients and 6.1% of non-diabetics had average glucose in the hypoglycaemic range (Table 2). On day 7 of the ICU stay, for those who remain in the ICU: 32.2% of diabetic and 12.4% of non-diabetic patients had average readings in the hyperglycaemic range; 1.4% of diabetic and 1.2% of non-diabetic patients had average glucose readings in the hypoglycaemic range. When examining minimum and maximum daily values, hyper- and hypoglycaemia occurred commonly on both day 1 and day 7 of the ICU stay.

**Table 2:** Percentage of patients experiencing abnormal blood glucose readings on day 1 and day 7 of the ICU stay (n = 19,694).

	Day 1		Day 7	
	Not diabetic (%)	Diabetic (%)	Not diabetic (%)	Diabetic (%)
Average < 80 mg/dL	6.1	2.0	1.2	1.4
Average > 180 mg/dL	9.3	28.6	12.4	32.2
Minimum < 80 mg/dL	13.7	21.0	6.5	11.6
Maximum > 180 mg/dL	27.9	61.1	28.4	59.3
Minimum < 50 mg/dL	1.2	3.0	0.4	1.9
Maximum > 200 mg/dL	18.8	49.5	19.6	48.3

Data are shown for average readings over 24 h and minimum or maximum reading during that day.

To test whether average glucose readings changed over the course of a patients stay, we compared the distribution of average glucose readings on day 1 with the distribution of average glucose readings on day 7, for the 2,759 first ICU stays which had at least one glucose reading on both these days. We used a Wilcoxon signed-rank test and found that there was no significant difference between the two distributions at the 0.05 significance level (*p* = 0.0675).

Similarly, we tested to see if the presence of hyperglycaemic and hypoglycaemic events varied over the course of the first week for the same group of patients. We used a chi-square test to separately test if the number of patients with at least one hyperglycaemic or hypoglycaemic events changed between day 1 and day 7. The test indicated that there was no significant difference for hyperglycaemic events between day 1 and day 7 at the 0.05 significance level (*p* = 0.286) for all patients. However, there was a statistically significant decrease in the number of patients with hypoglycaemic events (*p* = 0.036). These tests were performed separately on diabetic and non-diabetic patients. There were no significant changes in hyperglycaemic events for either group (diabetic hyperglycaemia *p* = 0.59, non-diabetic hyperglycaemia *p* = 0.36), and the change in hypoglycaemic events appears to be driven by observations of non-diabetic patients (diabetic hypoglycaemia *p* = 0.77, non-diabetic hypoglycaemia *p* = 0.011). The test for diabetic hypoglycaemia remains significant when applying the Bonferroni correction at the 5% significance level (critical *p* = 0.0125) for the four subgroup tests.

**Figure 3:**
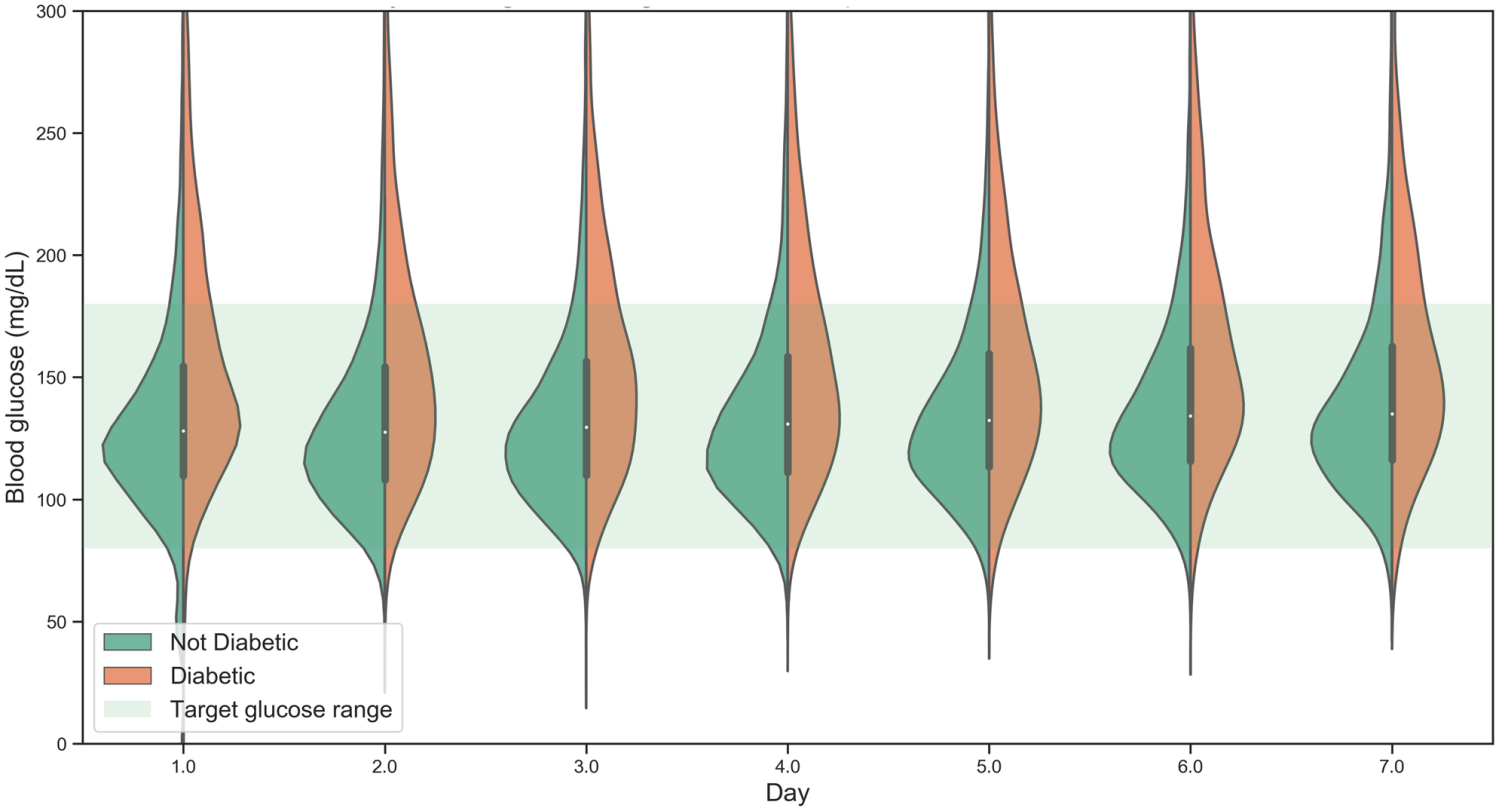
Average blood glucose for all patients (n = 19,694) in the first week of ICU admission. Diabetic patients are shown in orange and non-diabetic patients in green. The highlighted area marks the target glucose region between 180 and 80 mg/dL.

Patients who have abnormal glucose levels on any given day are likely to remain poorly controlled. On average the hazard ratio for hypoglycaemic patients becoming hypoglycaemic on the following day (relative to well-controlled patients) was 5.5. The equivalent hazard ratio for hyperglycaemic events is 4.1. This suggests there is a group of patients who are often poorly controlled. Average blood glucose for individual patients on any given ICU day ranged from approximately 40 mg/dL to greater than 300 mg/dL. This was true both at the time of ICU admission and on day 7 of ICU stay (Fig. 3).

### Insulin management

**Figure 4:**
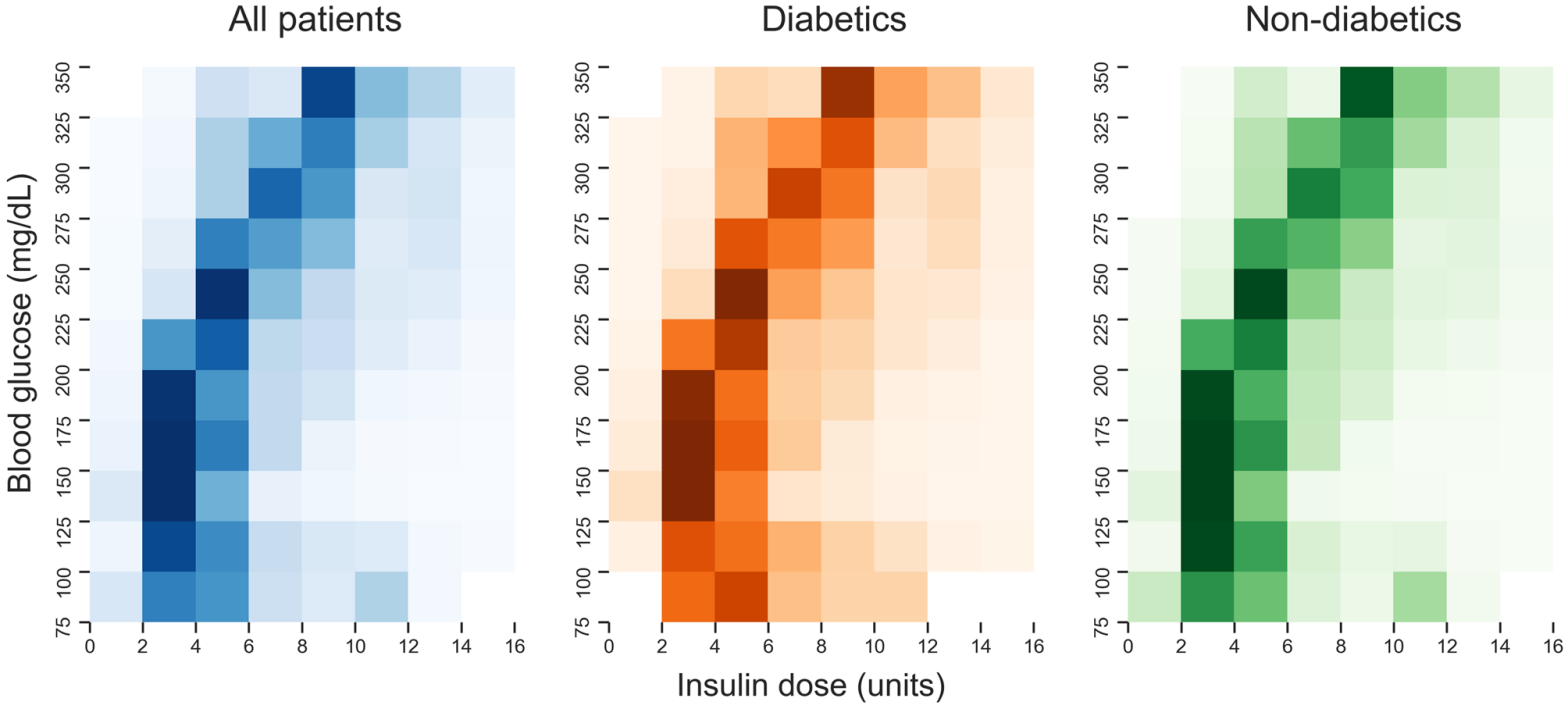
Short-acting insulin bolus dose distribution given glucose level. Heat map demonstrating the probability of receiving a dose of insulin given the blood glucose reading prior to insulin administration.

At any given blood glucose level, the doses of insulin administered to patients varied (Fig. 4). Figure 4 is a heat map of the probability of receiving a specific dose of insulin at a given glucose level. At times, patients with mild hyperglycaemia, ranging from 150 to 200 mg/dL, received anywhere from 0 to 16 units of boluses of short acting insulin. A similar broad range of insulin doses was observed for patients with severe hyperglycaemia, glucose ranging from 300 to 325 mg/dL. Of note, diabetic and non-diabetic patients received similar management strategies. To investigate whether diabetics received similar insulin doses at a given glucose level we regressed insulin dose on blood glucose, a diabetic status indicator and an interaction term using robust standard errors. The interaction term was a precisely estimated zero (coefficient = − 0.0004, *p* = 0.186) and the coefficient on the diabetic indicator was small but significant (coefficient = 0.1685, *p* = 0.003). Care should be taken when interpreting these standard errors because multiple observations come from the same patient and are correlated.

**Figure 5:**
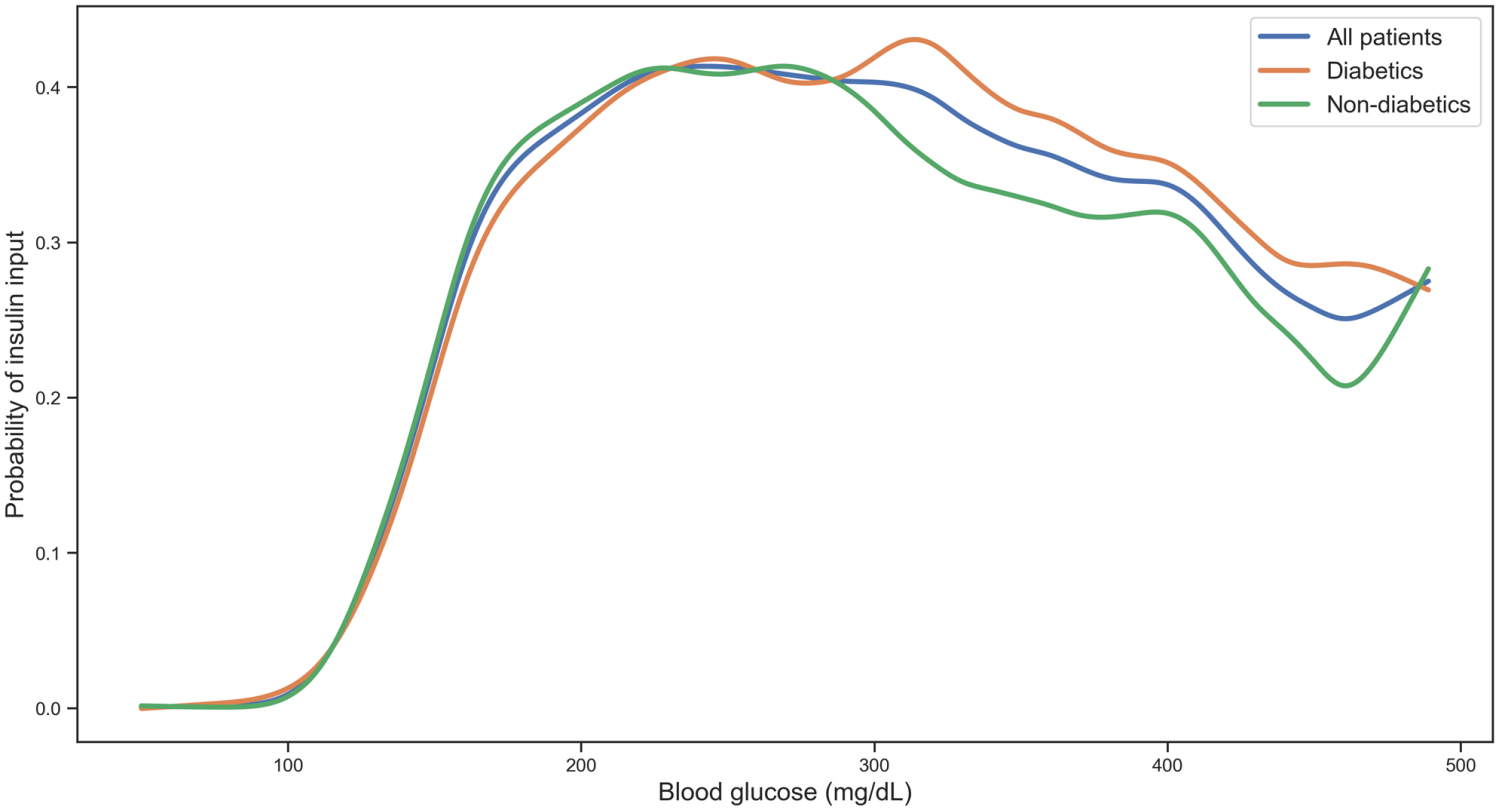
Probability of receiving short-acting insulin bolus after glucose measurement. Splines fitted to the proportion of glucose measurements which received an insulin input, for all patients, diabetics and non-diabetics.

Instead, we can use the regression results to compare predicted average doses for diabetics and non-diabetics. The regression predicts that for blood sugar reading of 200 mg/dL a diabetic would be given an average short-acting insulin dose of 4.72 units and a non-diabetic would be given 4.63 units. Similarly, at a blood sugar reading of 400 mg/dL, predicted average doses are 10.81 and 10.79 respectively. To investigate whether diabetics and non-diabetics also had the same likelihood of receiving a dose, we identified what proportion of glucose measurements received a short-acting insulin bolus at each glucose level for the two groups. Figure 5 shows that for a given glucose reading the probability of receiving a bolus of short-acting insulin is the same for diabetics and non-diabetics below 300 mg/dL, which constitute 95% of all bolus administrations. Above this level diabetics are more likely to receive insulin after a glucose measurement, other forms of insulin are likely used, and glucose measurements may be taken more frequently.

**Figure 6:**
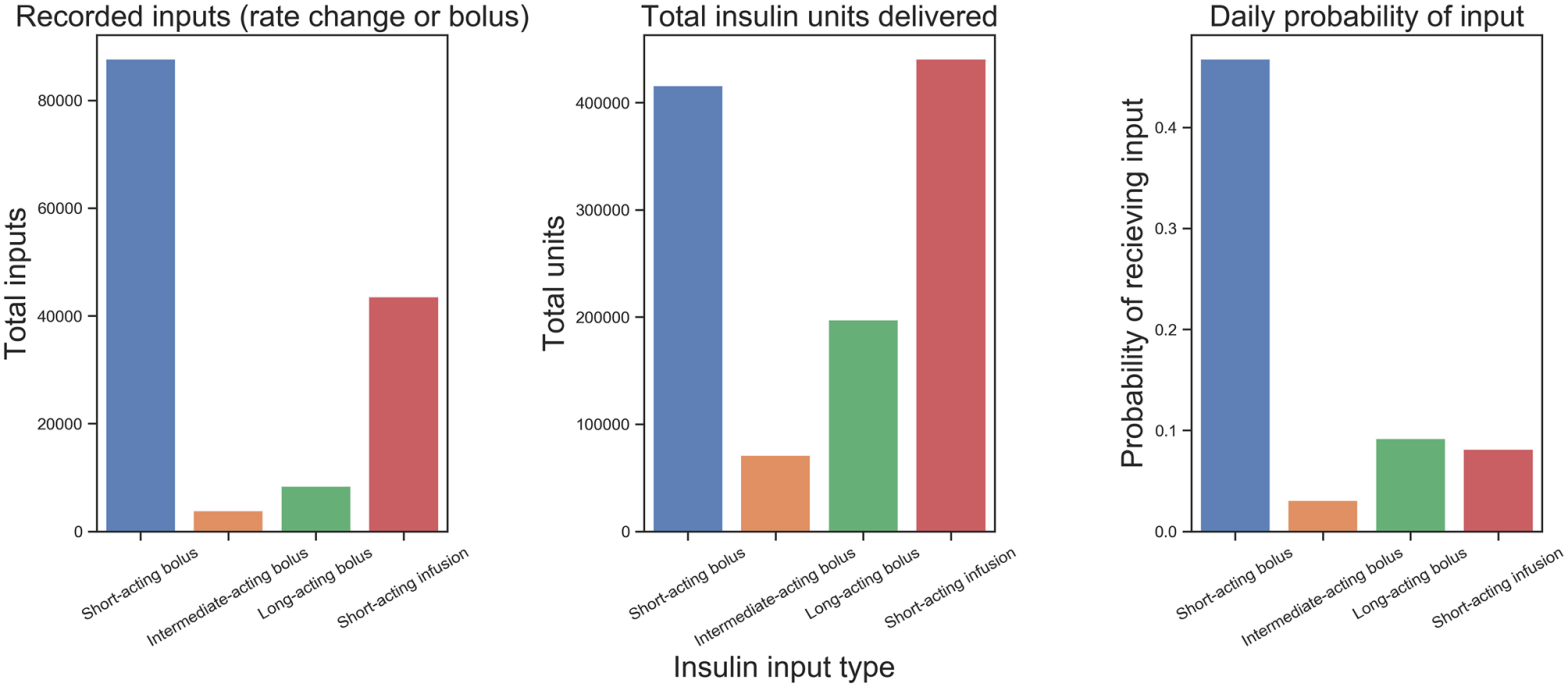
Probability of receiving insulin input on any given ICU day. Results are displayed as recorded inputs to electronic record (left), total units (center), and daily probability (right).

**Figure 7:**
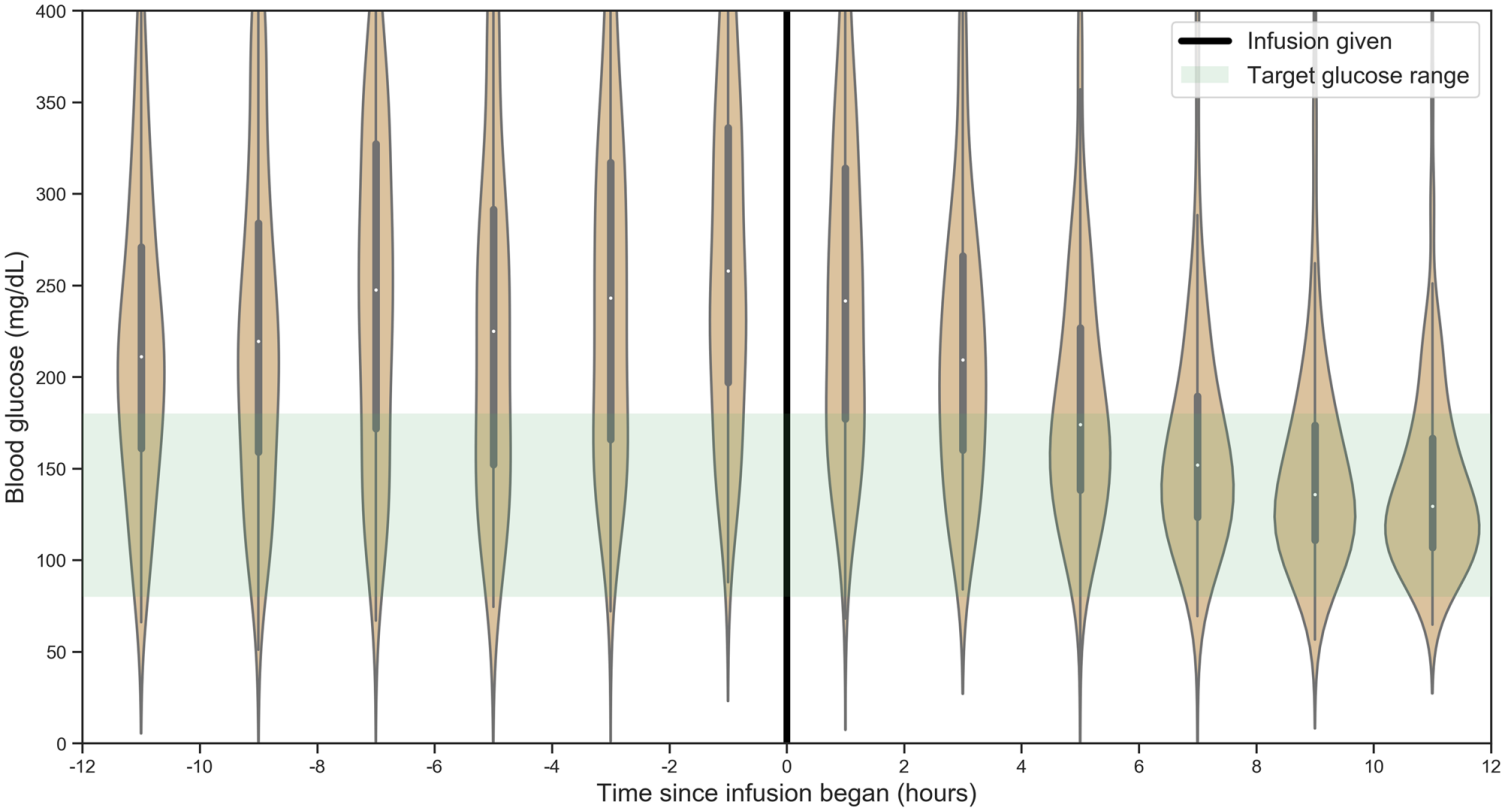
Average blood glucose for patients receiving insulin infusion (n = 371) during the 12 h preceding and following initiation of the infusion.

Overall, boluses of short acting insulin were utilized more commonly than infusions of short-acting insulin for controlling hyperglycaemia (Fig. 6). The proportion of units delivered with long-acting insulin remained consistent throughout the first week (22–24%), and the total amount of insulin per patient increased over the course of the first week. When utilized, insulin infusion was effective in controlling hyperglycaemia for the majority of patients receiving this therapy within 12 h of initiation (Fig. 7).

## Discussion

In a cohort of 19,694 ICU admissions to an academic medical centre, we provide the largest description to date of glucose management in the ICU. Patients demonstrated variable glycaemic responses during critical illness—both hypoglycaemia and hyperglycaemia were common at the time of admission and during subsequent days in the ICU. Additionally, successful management of abnormal glucose readings remains a challenge. Despite standardized approaches to care, our data demonstrate hyperglycaemic events were just as likely to occur 1 week into an ICU admission as at the time of ICU admission. A hypoglycaemic or hyperglycaemic event on a given day predicted the same event for that patient the next day.

Beyond the heterogeneous patient glycaemic profile and response to insulin during critical illness, approaches to short-acting insulin dosing varied. While some patients with a given blood glucose reading received little to no insulin, other patients may receive greater than 14 units for the same blood glucose. Comparing patients with and without diabetes, the use short acting insulin bolus dosing surprisingly did not differ between the two groups, despite known difference in sensitivity to exogenous insulin between these groups. Insulin infusion appears to be an effective means to control blood glucose among patients receiving this treatment, though it was not commonly used in this cohort. Limited use may be due to the labour-intensive nature of infusions, requiring dedicated intravenous access in patients who may require numerous simultaneous medication infusions and necessitating frequent monitoring to avoid hypoglycaemic events. Additionally, a shift in practice away from tight glycaemic control may have resulted in a perception that infusions introduce risk without clear benefit, resulting in underutilization of an effective means of controlling hyperglycaemia.

Our data provide an overview of glucose treatment strategies in a real-world ICU setting. While past studies have conflicted in their conclusions of the best treatment strategies, they have consistently suggested that both hypoglycaemic and hyperglycaemic episodes are associated with worse outcomes prompting protocolised approach to maintaining euglycaemia. Our data suggest that controlling glucose within a specified range is challenging for a significant proportion of patients. These data suggest that protocols which do not account for this heterogeneity among patients during critical illness, fail to adequately control glucose measurements in a significant proportion of patients. They provide an opportunity to understand the characteristics of patients at high risk for hypo- and hyperglycaemia and use predictive modelling to inform glucose management. These efforts may be informed by past studies which have identified risk factors for hypo- and hyperglycaemia in critically ill patients, including septic shock, mechanical ventilation, renal insufficiency, and increased severity-of-illness scores^11^.

These data must be interpreted in the context of our study design—a retrospective cohort study providing a description of a large observational dataset. While descriptive elements of glucose are valuable, we have not performed comparisons to understand if patient variability in glycaemic response to illness, or variability in insulin dosing, were independently associated with patient outcomes. Additionally, while we observed that patients received a wide variety of short-acting insulin bolus doses for any given blood glucose, this variability could in part be due to the clinical context—the illness severity and organ dysfunction which can affect the levels of circulating catecholamines and steroids, both endogenous and exogenous—and may therefore be appropriate variation. Unmeasured patient-level factors may also affect choice of insulin and explain some between-patient differences in insulin dosing at a given glucose level. The data used in our regression analysis are not strictly independent due to clustering at the patient level and autocorrelation. However, given autocorrelated data coefficient estimates remain unbiased, we focus on predicted insulin doses to draw our conclusion that diabetic and non-diabetic patients receive similar treatment^12^. MIMIC does not contain information on whether patients are diabetic and our method of flagging diabetes based on patient notes yields some false negatives and so may bias the effect size of diabetes downwards. Additionally, we do not have data on use of insulin prior to admission or an estimate of relative degree of insulin resistance—these are important confounders which may impact physician practice and insulin dosing. Given the variability in glucose control observed, it is also possible that the protocols guiding clinical care, rather than patient or physician-level factors, are responsible for inadequate glucose control in some instances. Finally, the data analysed are from the years 2008–2012. While the routine approach to insulin bolus dosing has not been modified significantly since that time, current critical care practices may have changed in measurable ways that are not reflected in the data.

The curation of the database built for this study from MIMIC may now facilitate the design of predictive models and development of individualized and more precise treatment strategies. A universal protocol for glucose management, which is routine practice for most ICUs worldwide, appears impractical and ineffective, based on our data.

## Methods

We performed a retrospective cohort study using the Medical Information Mart for Intensive Care III v1.4 (MIMIC) database^13^. MIMIC-III contains data on greater than 60,000 ICU admissions between 2001 and 2012 at Beth Israel Deaconess Medical Center (BIDMC) in Boston, Massachusetts. BIDMC is a large urban teaching hospital affiliated with Harvard Medical School. The data in MIMIC-III has been de-identified, and the institutional review boards of the Massachusetts Institute of Technology (No. 0403000206) and Beth Israel Deaconess Medical Center (2001-P-001699/14) both approved the use of the database for research. All data analysis and reporting has been performed in accordance with institutional guidelines and regulations. As a de-identified, publicly available database, patient consent is not required for the use of MIMIC-III by investigators.

Decisions surrounding insulin treatments in the ICU are made by a multidisciplinary team that includes attending physicians, resident physicians, nurses, and pharmacists. Insulin order options are standardized in the electronic medical record system and insulin management follows a protocol within the ICU. Glucose levels are monitored every 4 h upon ICU admission for most patients receiving bolus insulin, and the target glucose range is 140–180 mg/dL. For patients with glucose levels greater than 180 mg/dL, bolus of short-acting insulin is administered beginning with a weight-based sliding scale, such that insulin dose increases incrementally as blood glucose increases above specific thresholds (e.g. 200, 250, 300, 350 mg/dL). Initial insulin doses along that scale may be modified at the ICU physician’s discretion and may incorporate a patient’s prior known insulin requirements or diabetic status. For patients who do not remain well controlled on a given sliding scale, doses may be increased at the physician’s discretion. Insulin infusions are not routinely used at the outset of critical illness, in the absence of diabetic ketoacidosis, unless a patient’s glucose levels cannot be controlled using a sliding scale of boluses of short-acting insulin. Intermediate and long acting insulin are not routinely used in the ICU although may be used for patients with high baseline insulin requirements or new significant insulin requirements in the ICU.

**Figure 8:**
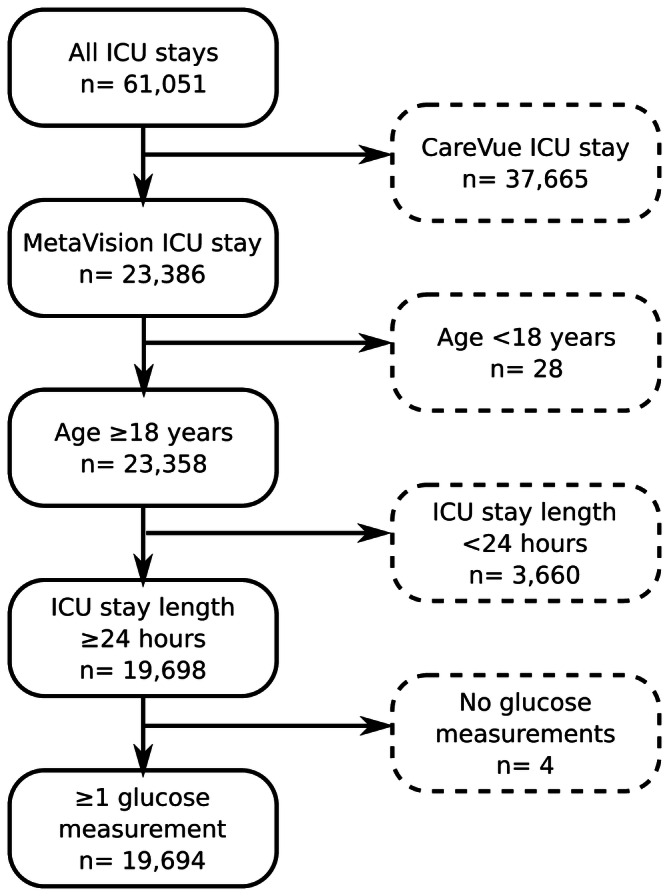
Exclusion process for ICU stays.

The study cohort consists of ICU admissions from the MetaVision clinical information system which covers 2008–2012. To be included in the analysis, ICU stays needed to meet the following criteria: patients aged 18 or over, ICU length-of-stay 24 h or greater and at least one glucose measurement during the patient’s ICU admission (Fig. 8). Glucose measurements, insulin inputs and dextrose inputs were extracted for each ICU admission included in the cohort. Each event category was filtered to remove: entries flagged as errors; duplicates; events occurring outside the ICU; and entries with physiologically implausible values. Diabetic status was determined by physician notes, which included keywords such as “diabetes,” “diabetes mellitus,” or “insulin dependent.” Average daily glucose measurements were time-weighted, so that multiple measurements in quick succession did not distort the average.

We matched glucose measurements and insulin inputs to create measurement-input pairs. In MIMIC, events are not explicitly associated with one another. However, in clinical practice, one expects that any bolus of short-acting insulin input would be linked to a recent glucose measurement. To control for insulin used for reasons other than blood sugar control, we excluded insulin inputs for 298 patients with a primary diagnosis of diabetic ketoacidosis, beta blocker overdose, or hyperkalaemia. We also excluded 1,204 boluses of short-acting insulin given within 1 h of a calcium gluconate input, a treatment for hyperkalaemia. Measurements were linked by defining a time window around a short-acting insulin bolus administration and identifying the most recent glucose measurement which occurred within that window. The window limits were defined as 60 min prior to the insulin input (nurses may only enter timestamps to the nearest hour) and 10 min after the insulin input. While one might expect all glucose measurements to occur before the insulin input, hospital staff may inadvertently enter them in the opposite order. Short-acting insulin bolus inputs for which no matching measurement was found were discarded. Unmatched measurements were similar to matched measurements on proportion given to diabetic patients, insulin dose, nearest glucose measurement and time of stay. We defined hyperglycaemia as glucose > 180 mg/dL and hypoglycaemia as glucose < 80 mg/dL. The initial queries were performed on PostgreSQL and further data cleaning and data visualization were done on Python 3.7 through packages widely used in data science.

### Data availability

As mentioned before, the data that support our conclusions employs the MIMIC-III data. It is a widely used dataset in the analysis of real-world health data^14,15^. Researchers need to request permission to get accession and data available as a collection of comma separated value (CSV) files. Further instructions are available in the MIMIC Code Repository^16^.

The data extraction and visualization is available online as a jupyter notebook in the MIMIC Code Repository^16^.
